# Facile green synthesis of selenium nanoparticles using olive (*Olea europaea*) leaf extract and their antimicrobial and antibiofilm properties

**DOI:** 10.1038/s41598-026-47329-5

**Published:** 2026-05-16

**Authors:** Sama E. Hassan, Nada M. Khedr, Esraa M. Omran, Sara R. Mohamed, Nourhan A. Gemail, Abdelrahman A. Elsharawy, Dina M. Baraka, Rasha Mohammad Fathy

**Affiliations:** 1 Microbiology and Biochemistry Programme, Faculty of Science, Benha University, Cairo, Egypt; 2 Botany and Microbiology Department, Faculty of Science, Benha University, Cairo, Egypt; 3 Drug Radiation Research Department, National Center for Radiation Research and Technology (NCRRT), Egyptian Atomic Energy Authority, Cairo, Egypt

**Keywords:** Olive, *Olea europaea*, Selenium nanoparticles, *C. albicans*, Antimicrobial, Antibiofilm, Biotechnology, Microbiology, Nanoscience and technology

## Abstract

The spread of antibiotic-resistant microorganisms is increasing, posing a significant health threat to humans. The world is now using nanoparticles, particularly through green synthesis, as an effective alternative to traditional antimicrobials. Selenium nanoparticles (Se NPs) were produced from the *Olea europaea* plant extract and have been characterized using UV-Visible, FTIR, SEM, EDX, zeta potential, and HR-TEM. The antimicrobial effectiveness of Se NPs was evaluated against several microbial strains, including *Staphylococcus aureus*, *Escherichia coli*,* Klebsiella pneumoniae*, *Citrobacter freundii*, and *Candida albicans*, and its impact on their biofilm formation was tested. The UV-Vis spectrophotometer results displayed the highest absorption at 380 nm, indicating the synthesis of selenium nanoparticles. The biosynthesized Se NPs were uniform, well-dispersed, quasi-spherical in shape, and with particle sizes ranging from 2.60 to 4.75 nm. The antimicrobial activity analysis of Se NPs showed that *C. albicans* was mostly inhibited, recording an inhibition zone diameter of 35.98 ± 0.65 mm at 150 µg/mL. The antibiofilm assessment represented a 59.04% inhibition of *C. albicans* biofilm. In conclusion, Se NPs have proven to be economical, less toxic, and environmentally friendly antimicrobial agents for preventing and treating many bacterial and fungal infections.

**Supplementary Information:**

The online version contains supplementary material available at 10.1038/s41598-026-47329-5.

## Introduction

Antibiotic resistance is a health threat and is closely related to morbidity and mortality rates. The unnecessary overuse of broad-spectrum antibiotics has caused the emergence of bacterial resistance^1^. Resistance mainly increases through genetic mutations and horizontal gene transfer, allowing bacteria to survive antibiotic exposure^2^. Structural differences in bacteria affect resistance strategies. In Gram-negative bacteria, the lipopolysaccharide layer provides an intrinsic barrier to antibiotics, make them more resistant to antibiotics than Gram negative bacteria^3^. The *Mycobacterium* outer membrane is composed of lipids, so hydrophobic drugs can further enter the cell. Resistance to cell wall-targeting drugs, such as β-lactams, occurs in bacteria that lack cell walls, such as *Mycoplasma* (del Olmo and Andreu, 2025). On the other hand, Gram-positive bacteria lack an outer membrane, so antibiotic entrance into the cell is more easier^4^. Resistance also develops through two mechanisms: drug degradation (e.g., β-lactamase activity) or drug modification by chemical group transfer^5^.

Olives (*Olea europaea*) are powerful antioxidants that neutralize free radicals that contribute to many diseases (Guo et al., 2018). The main fatty acid in olive oil is a monounsaturated fat that increase good cholesterol. It prevents the formation of inflammatory enzymes that lead to diseases like arthritis and diabetes mellitus and help in avoiding the combination of chronic oxidative stress^6^. Some olives provide probiotics that maintain gut health and support the microbiome. Olives contain vitamin E, which is good for healthy skin and keeps the balance of pH^7^.

The nanoparticles are employed for all the aforementioned purposes, the metallic nanoparticles are considered to be most promising because they contain notable antibacterial capabilities due to their large surface area to volume ratio, which are significant to investigators due to the rising microbial resistance towards metal ions, antibiotics, and the appearance of resistant^8^^,9^. Nanoparticles (NPs) can be utilized as bactericidal or as carriers for other therapeutic drugs^10^. Nanomaterials have also shown promising results in overcoming biofilm-associated resistance^11^. Moreover, nanoparticles are used in industrial sectors viz., food safety, medicine and other fields^12,13^.

Selenium is an essential nutrient for metabolic processes in humans, animals, and plants. It is also important for increasing immune response in humans, reproduction in animals, and plant growth^14^. Selenium exists in two forms (inorganic and organic). Organic forms are employed in biological uses such as antioxidant, antibacterial, anticancer, antiviral, antidiabetic, and depressive properties, as well as a nutritional source. It also acts as a modulator in metabolism, DNA synthesis, thyroid hormone regulation, and protection from infections^15^. The advised daily consumption of selenium is around 60 µg for women and 70 µg for males^16^. Se NPs, having diameters ranging between 5 and 200 nm, have gained great attention in recent years (Galić et al., 2023). Se NPs can be formed by three methods: biological, physical, and chemical. Biological synthesis involves the reduction of selenium using bio-agents such as microorganisms or plant extracts, transforming them into selenium nanoparticles^15^. The biological method (green synthesis) is mostly preferred, as it is less toxic, safer, environmentally friendly, and economically viable^17^.

Selenium nanoparticles (Se NPs) have sparked worldwide interest due to their high absorption, biological activity, and low toxicity compared to their Se-based materials. Se NPs have been widely recognized to have a broad antibacterial and antifungal action^15^. Their antimicrobial activity may be linked to the excessive formation of reactive oxygen species (ROS)^18^, which results in disruption of signaling pathways ending with microbial cell damage^10^. These essential characteristics have motivated researchers to assess the potential of Se NPs as a strategy for combating multidrug-resistant bacteria and other microbial diseases^19^. Nonetheless, further investigations on the antibacterial effect of Se NPs show that the variety in synthesis techniques and particle size affect their biological activity^20^. Furthermore, the synthesis method significantly affects the toxicity of Se NPs. Thus, biogenic Se NPs are more biocompatible and less toxic^21^. Green synthesis techniques have numerous advantages, including lower ecological effects, low-cost, and biological compatibility, which make them a promising option for sustainable nanomaterial synthesis^22^.

The present study aimed to biosynthesize Se NPs using aqueous extract of *Olea europaea *leaves, characterize the prepared Se NPs, and determine their antimicrobial and antibiofilm effects.

## Materials and methods

### Materials

Sodium selenite (Na_2_SeO_3_) was purchased from Advent Company Mumbai. Ascorbic acid and Polyvinylpyrrolidone were purchased from a German company Lanxess. The nutrient broth medium was purchased from Himedia Laboratories Pvt. Ltd. India.

### Plant sample collection

**Fig. 1:**
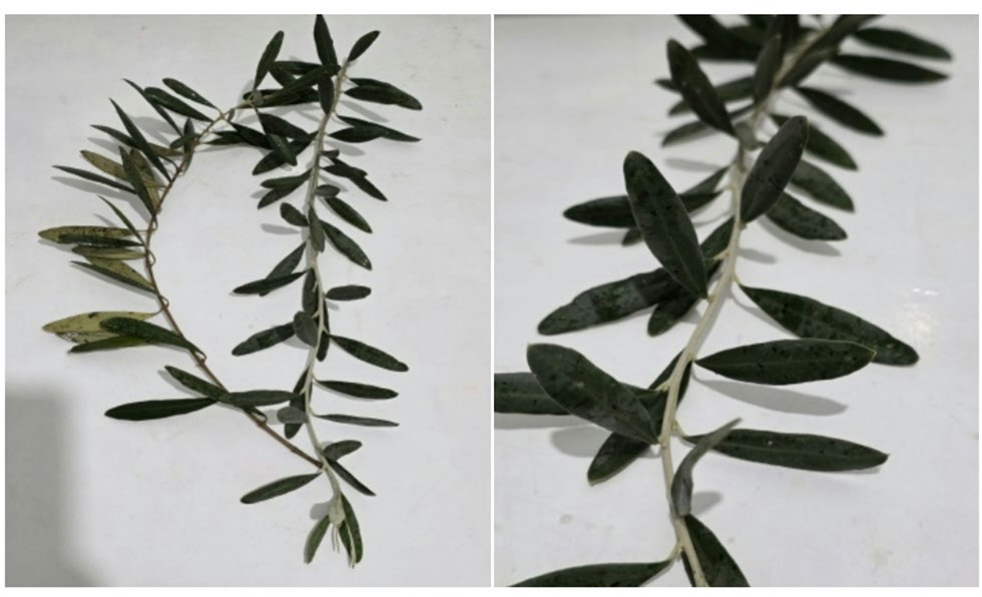
The collected *Olea europaea* leaves.

The olive *Olea europaea* leaves were collected from local gardens in Shebin El Qanater city, Al-Qalyubia Governorate, Egypt in 2025. Location is (30° 18’ 45.68” N, 31° 19’ 12.65” E) stands at an average elevation of 20 m (66 feet) above sea level (Fig. 1). To get rid of dust, the leaves were cleaned with tap water. Olive leaves were dried at room temperature and chopped into fine cuts.

### Preparation of aqueous *Olea europaea* leaf extract

The *Olea europaea* leaf extract was prepared according to^23^ by placing 50 g of dried fine cut with 500 mL of deionized water. The mixture was then heated up to 60 °C for 30 min. The initially colorless solution was turned yellow. The extract was filtered using a muslin cloth and was filtered by No. 1 filter paper. The extract was stored at – 4 °C to be used for further experiments.

### Green synthesis of selenium nanoparticles using *Olea europaea* leaf extract

**Fig. 2:**
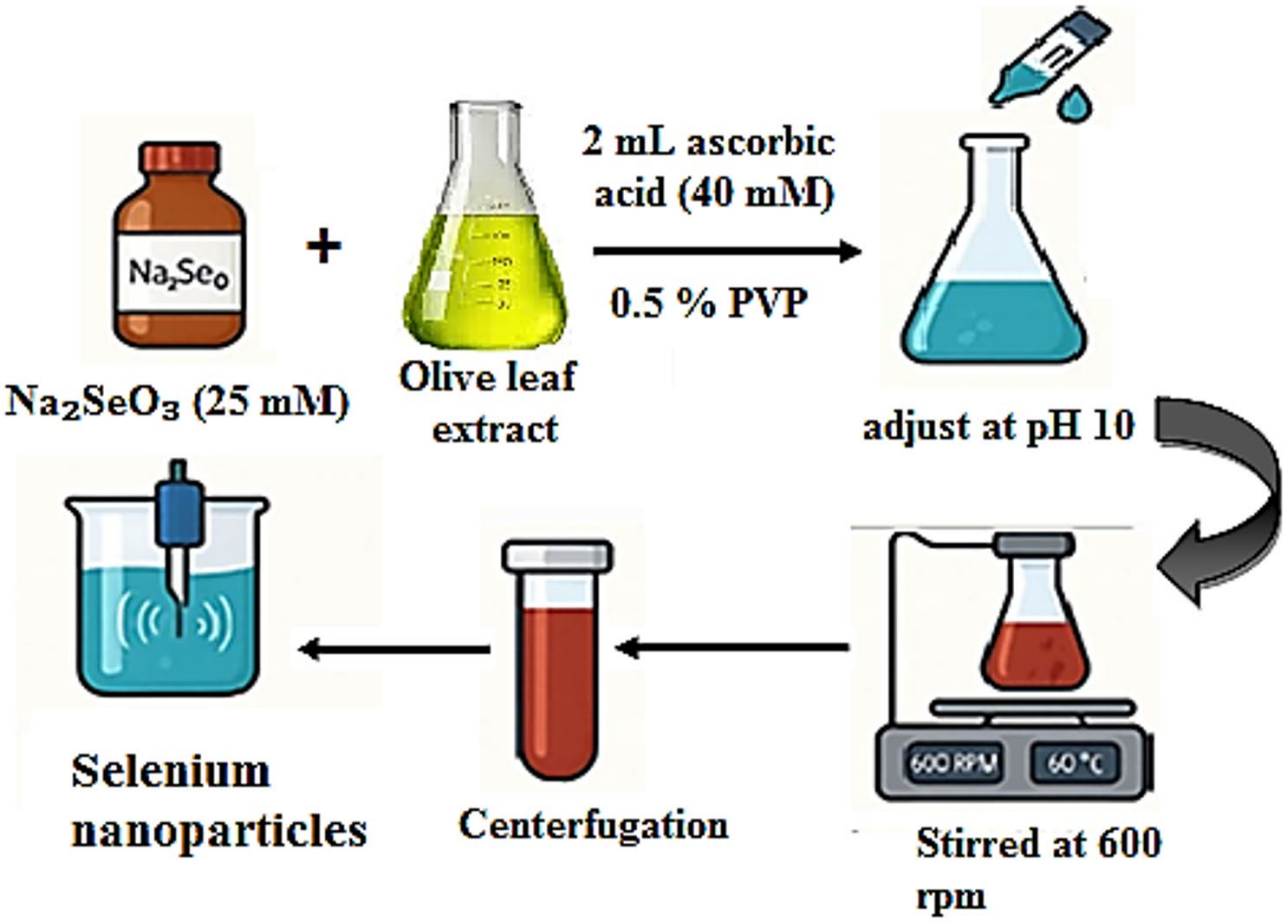
Biological synthesis of Se NPs using olive leaf extract.

25 mM solution of sodium selenite (Na₂SeO₃) was prepared, and then added to the *Olea europaea* leaf extract at a ratio of 4:10 (v/v). Additionally, 2 mL of 40 mM ascorbic acid and 0.5% of PVP were added. The pH was adjusted to 10 with 1 M sodium hydroxide. The mixture was stirred at 600 rpm and 60 °C for 2 h. The mixture was centrifuged at 10,000 rpm for 15 min, the supernatant was discarded, and the pellets were purified by washing them three times with distilled water. Finally, the pellets were dried to be characterized and resuspended in deionized water at the concentrations (µg/mL) required for biological assays, followed by ultrasonication for 30 min^24^. The biological synthesis of Se NPs was summarized in Fig. 2.

### Characterization of the biosynthesized Se NPs

The UV-Visible spectrophotometer (T60 UV-Vis-UK) was utilized to analyze Se NPs. The Fourier-transform infrared spectroscopy (FT-IR) examination was accomplished on a Bruker-Germany FTIR-Vertex 70 apparatus using potassium bromide (KBr) pellets flattened with dried samples. Zeta potential (DLS-PSS-NICOMP 80-ZLS particle sizing system, St. Barbara, CA, USA) was utilized to determine the particle charge and distribution. The X-ray diffraction examination was performed with the XRD 6000 series, counting residual crystallite size/lattice and crystal nature (Shimadzu apparatus employing Cu-K(α), λ = 1.5418 Å and nickel filter Shimadzu Scientific Instruments (SSI), Tokyo, Japan). Scanning electron microscopy (SEM, ZEISS-EVO 15-UK) was used to study the nanoparticles morphology. The content and concentration of elements in nanoparticles were determined by energy dispersive X-ray analysis (EDX). The high-resolution transmission electron microscope (HR-TEM) micrographs of Se NPS were studied using a JEM-2100 plus (JEOL) with an accelerating voltage of 200 kV.

### The antimicrobial activity of biosynthesized Se NPs against various pathogenic strains

The agar well diffusion procedure was used to evaluate the antimicrobial activity of the biosynthesized Se NPs against the pathogenic strains, *Staphylococcus aureus* ATCC 6538, *Escherichia coli* ATCC 25922, *Klebsiella pneumoniae* ATCC 13883, *Citrobacter freundii* ATCC 8090, and *Candida albicans* ATCC 2091 that were kindly procured from the Drug Microbiology Lab, National Center for Radiation Research and Technology, Egyptian Atomic Energy Authority as described by^25^. Pure colonies of the tested strains were inoculated in nutrient broth for 24 h. To adjust 0.5 McFarland concentration that is equal to (1–2) × 10^8^ CFU/mL, the suspension’s OD must fall within the target range of 0.08 to 0.13. A100 µL of each microbial strain, *S. aureus*,* E. coli*,* K. pneumoniae*,* C. freundii*, and *C. albicans* culture broth was finely spread by sterile cotton swab on the surface of nutrient agar plates. Four wells with a diameter of 6 mm were made using sterile micro tips (back side) into each plate for dipping the Se NPs and plant extract (negative control). The concentrations of Se NPs of 150, 75, and 50 µg/mL were prepared (resuspended in water with ultrasonication for 30 min), and 100 µL of each concentration was loaded into separate wells. One well in each plate was loaded with 100 µL of the plant extract to compare its activity. Amikacin 30 µg was used as a positive control. The Petri plates were incubated at 37 °C for 24 h. After incubation, clear inhibition zone diameters around the wells were estimated in mm.

### Determination of minimum inhibitory concentration (MIC), minimum bactericidal concentration (MBC) minimum fungicidal concentration (MFC)

MIC, MBC, and MFC were assessed using the methods of^17^. The inoculums (10^4^ CFU/mL) were inoculated on nutrient agar plates and then Se NPs at concentrations ranging from 3.125 to 100 µL were applied. The plates were subsequently incubated for 24 h at 37 ˚C. The lowest dosages at which no bacterial growth was discernible on the surface of agar plates were known as the minimum inhibitory concentrations, or MICs. The MBC is defined as the lowest concentration of an antimicrobial agent that kills 99.9% of a specific bacterium. Also, MFC is defined as the lowest concentration of antimicrobial agent resulting in the death of 99.9% of fungi.

### The antibiofilm activity of the biosynthesized Se NPs towards pathogenic strains

The test tube method was applied to assess the antibiofilm capability of Se NPs according to Kodous et al.^26^. Five milliliters of the nutrient broth were used to prepare test tubes containing *S. aureus*,* E. coli*,* K. pneumoniae*,* C. freundii*, and *C. albicans.* 10 µL of 0.5 McFarland (1 × 10^8^ CFU/mL) of the examined strains was inoculated into 5 mL of the nutrient broth tubes. 5 mL of Se NPs at a concentration of 150 µg/mL was added. The control tubes were prepared in the same way, substituting the same amount of Se NPs with distilled H_2_O. The test tubes were incubated at 37 °C for 24 h. After incubation, the media of the tubes were discarded, and the tubes were rinsed with phosphate buffer saline (PBS, pH 7) and allowed to dry. 5.0 mL of sodium acetate (3.0%) was used to stabilize the adhering bacteria for 10 min, and then the tubes were rinsed with deionized water. The biofilms were stained with crystal violet (0.1%) for 10 min. The stain was discarded and washed with deionized water to remove the remaining stain. 2.0 mL of ethanol was added to each tube to dissolve the crystal violet stain. The biofilms were evaluated quantitatively using a UV–Vis spectrophotometer at λ꞊570 nm. The proportion of biofilm inhibition was assessed using the following Equation^27^.

1 $${\text{Biofilm~inhibition~\% ~}}={\mathrm{~}}\frac{{{\mathrm{O}}{{\mathrm{D}}_{{\mathrm{c~}}}} - {\mathrm{~O}}{{\mathrm{D}}_{\mathrm{t}}}}}{{{\mathrm{O}}{{\mathrm{D}}_{\mathrm{c}}}}}{\mathrm{~}} \times 100{\mathrm{~}}$$

Where OD_c_ is the optical density of the control (with no Se NPs) and OD_t_ is the optical density of the Se NPs-treated.

### Statistical analysis

Antimicrobial efficiency tests were conducted in triplicates, with findings reported as mean ± SD. Statistical analyses were performed using SPSS (IBM) software (V24), including one-way ANOVA at *P* < 0.05 and Duncan’s multiple range analysis.

## Results and discussion

### Synthesis of Se NPs

After preparing the aqueous *Olea europaea* leaf extract, it was mixed with sodium selenite solution (25mM) in a 4:10 (v/v) proportion, and then the mixture was stirred for 2 h. A change in the solution color to dark red showed the production of Se NPs. Ascorbic acid was used as a reducing agent to reduce the sodium selenite. The most commonly used reducing agent is ascorbic acid because the reaction may be performed at ambient temperature in water and the ascorbic acid is affordable and non-harmful^28^. Moreover, PVP acts as a partial stabilizing agent^29^ in conjunction with the plant extract, allowing for greater stability of the synthesized Se NPs and increasing their range of biological applications. The color of the solution changed to dark red, indicating the synthesis of Se NPs. This color change is caused by the excitation of surface Plasmon resonance, which confirms the reduction of sodium selenite to elemental selenium.

### Characterization of Se NPs

#### UV- visible spectroscopy

**Fig. 3:**
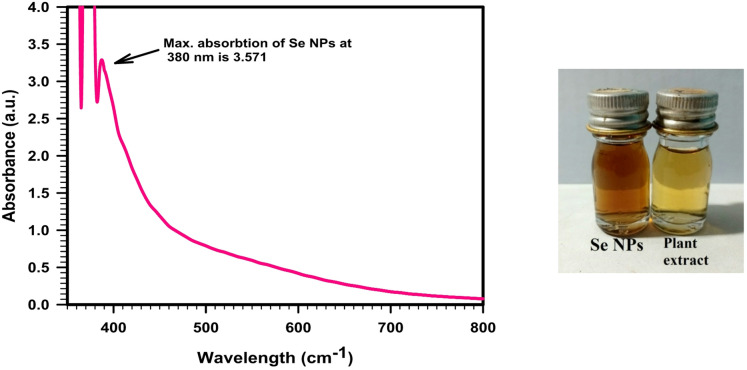
UV–visible spectrum of the green synthesized Se NPs using *Olea europaea* leaf extract.

The biosynthesized Se NPs were analyzed using UV-visible spectroscopy to assess their optical properties. Biogenic synthesis of Se NPs was characterized using the UV–Visible spectrum in the range of 200–800 nm. Figure 3 depicts the color changes after nanoparticles formation, where the colloidal solution was colorless at first but turned light red with the formation of Se NPs. The colloidal solution displays a maximum absorption of 3.571 at λ = 380 nm certainly demonstrates the production of Se NPs. Color changes from pale yellow to brick red, light pink, or deep red indicates the production of Se NPs^30^.

Biomolecules such as proteins, polysaccharides, and phytochemicals (polyphenols, flavonoids, terpenoids, alkaloids, and saponins) are abundant in plants and act as stabilizing, capping, and reducing agents for the environmentally friendly synthesis of nanoparticles. Numerous plant species’ flowers, roots, peels, fruits, seeds, and leaves have been studied as potential sources for nanoparticles synthesis^31^. However, due to their hydroxyl and ketone groups, which can bind to metals and exhibit chelating properties, it is known that the type, concentration, and source of phytochemicals in the plant extract, as well as the extraction process, influence the properties of the nanostructure^32^. As reported by previous work, Se NPs synthesized using *Melia azedarach* aqueous leaf extract showed promising antifungal efficiency^33^. Moreover *Portulaca oleracea*-based green Se NPs revealed antibacterial, antiviral, and mosquitocidal activities^34^.

#### Fourier Transform Infrared spectroscopy (FTIR) analysis

**Fig. 4:**
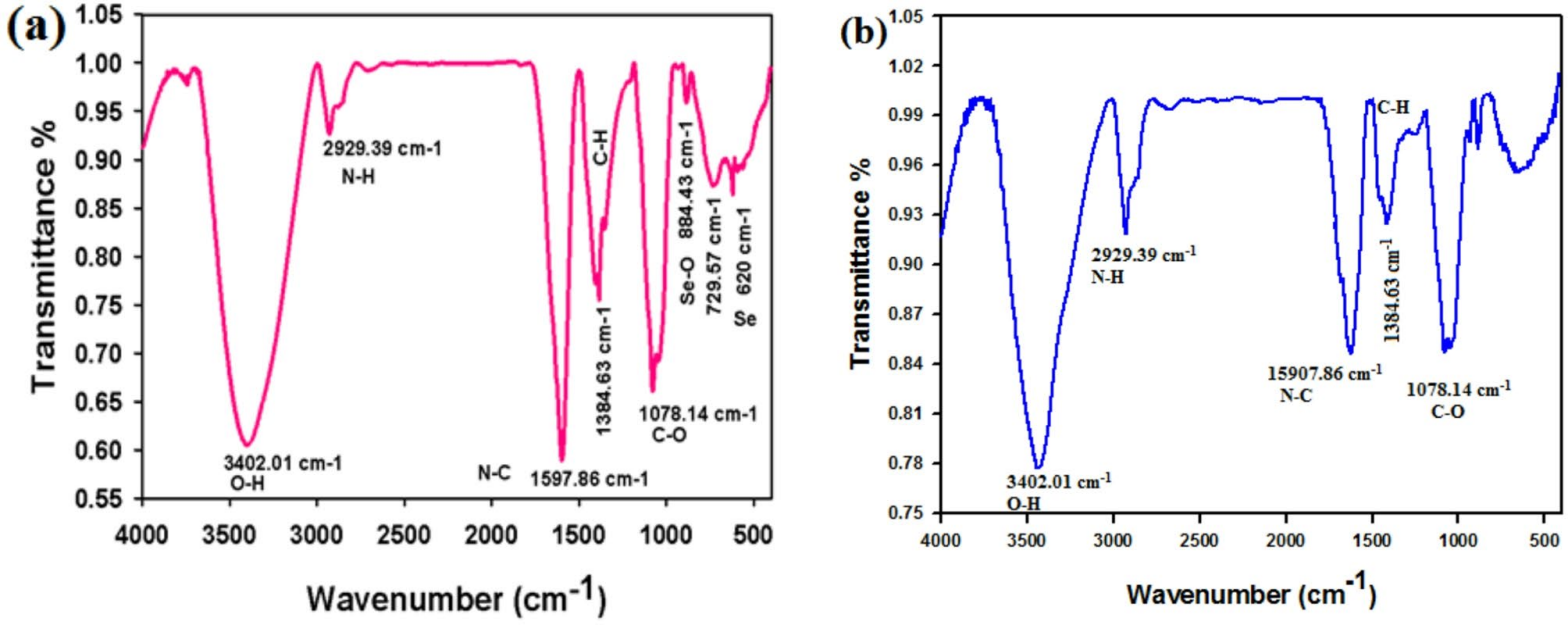
FTIR spectrum of (a) *Olea europaea* leaf extract and (b) the biosynthesized Se NPs.

The FTIR characterization of Se NPs was conducted by a Fourier Transform Infrared (FTIR) spectrometer at the range between 4000 and 400 cm-¹. As shown in Fig. 4a, the Se NPs show several different peaks at 3402.01, 2929.39, 1597.86, 1384.63, and 1078.14 cm^-1^. The peak at 3402.01 cm^-1^ refers to the O-H stretching band is for the free alcoholic group, intermolecular bond, or amine group (N-H stretching)^35^. The peak at 2929.39 cm^-1^ is associated with the C–H stretch band of alkenes^24^. The peak at 1597.86 cm^-1^ contributes to N–C and C–C stretching band, which may be due to the presence of protein extract^36^. The band at 1384.63 cm^-1^ is for C-H asymmetric bending in groups of CH_2_ and CH_3_ (Fritea et al., 2017). The C-O stretching in an amino acid produces a band at 1078.14 cm^-1^. These functional groups, found within the plant’s phytochemicals, such as polyphenols, flavonoids, proteins, and organic acids, bind to the metal surface to stabilize Se NPs. Figure 4b shows the same peaks found in the *Olea europaea* leaf extract sample, in addition to some other beaks that appeared at 884.43, 729.57, and 620 cm^-1^. The spectra exhibit a pronounced peak at 884.43 cm^-1^ corresponding to Se-O stretching within the sodium selenite fingerprint region^37^. The band at 729.57 cm^-1^ may indicate stretching vibrations in metal-oxygen bonds^38^. The fingerprint region at 620.04 cm^-1^ contains a broad, strong band that corresponds to elemental selenium stretching vibrations^39^.

#### X-ray diffraction analysis

**Fig. 5:**
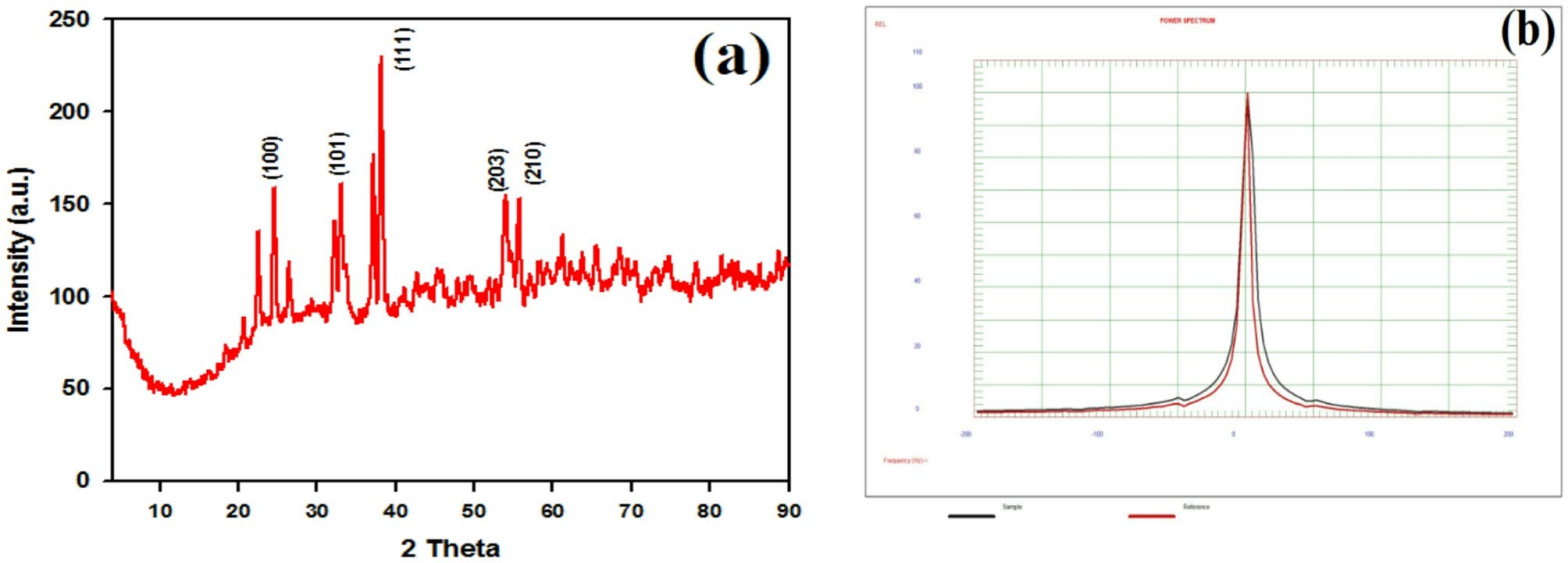
(a) XRD and (b) Zeta potential of Se NPs.

The Crystalline structure and purity of the biosynthesized Se NPs was determined using X-ray diffractometer (XRD) (Fig. 5a). The strongest diffraction peaks appear at 2θ values of 22.54, 32.24°, 33.05°, 33.60°, 38.16°, 42.73, 53.97°, and 55.67° that are identified as 100, 101, 110, 122, 111, 201, 203, and 210 reflections^40^^,41^ in accordance with the JCPDS card No. 06-362. Sharp peaks indicate good crystallite development, and the positions of the peaks confirm the synthesis of pure Se NPs. The results confirm the crystalline structure of the Se NPs.

#### Zeta potential

The zeta potential of the prepared Se NPs was evaluated to determine their surface charge characteristics. The analysis reveals that the Se NPs exhibit an average zeta potential of + 4.46 mV as shown in Fig. 5b. Nanoparticles with zeta potentials ranging from − 10 to + 10 mV are categorized as essentially neutral (Clogston and Patri, 2011) whereas those with zeta potentials higher than + 30 mV or lower than − 30 mV are termed highly cationic and strongly anionic^17^. A large zeta potential ( > ± 30 mV) promotes colloidal stability through strong electrostatic repulsion between particles, preventing aggregation and flocculation. While lower zeta potential ( < ± 30 mV) signifies instability as electrostatic forces weaken, permitting attractive van der Waals forces to dominate and promote particle clumping^42^.

#### Scanning electron microscopic analysis

Selenium nanoparticles synthesized using *Olea europaea* leaf extract can be seen embedded in a matrix. The scanning electron microscope images, obtained at different magnifications using secondary electrons (SE1) at an accelerating voltage of 22.00 kV, provide a detailed view of the surface morphology of the sample. The Se NPs display an irregular, flaky to amorphous morphology, forming visible clusters across the substrate. The background surface appears relatively smooth, with noticeable coverage by the particle structures. These observed features reflect a polydisperse distribution of the particles and provide insight into the structural and surface properties of the sample at the microscopic level.

**Fig. 6:**
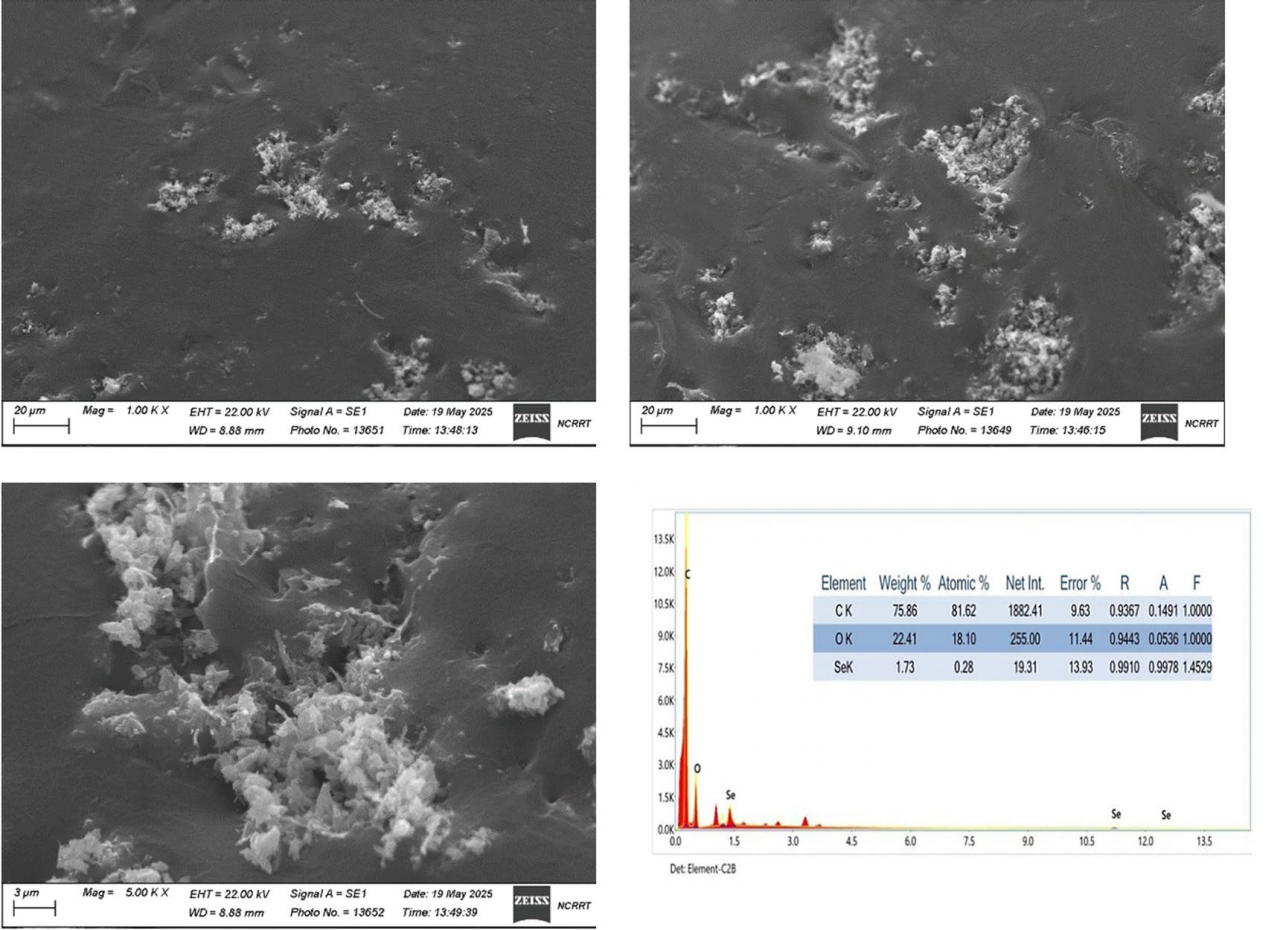
SEM–EDX of Se NPs at different magnifications.

EDX analysis was performed to determine the concentration and composition of the elements in the biosynthesized Se NPs. Figure 6 shows absorption peaks for green synthesized Se NPs from *Olea europaea* that are undergoing quantitative examination. The highest intensity was obtained by carbon (C), followed by oxygen and finally selenium. The corresponding weights of carbon, oxygen, and selenium are 75.86%, 22.41%, and 1.733%, respectively. The presence of carbon, oxygen in the elemental structure of Se NPs may be corresponding to the components of *Olea europaea*.

In accordance with previous works^43^, prepared rods shape Se NPs with a size in the range of 74.29 nm. SEM analysis of Se NPs synthesized using *Hibiscus rosa-sinensis* leaf extract revealed spherical Se NPs with an average diameter of 47.6 nm^44^. The Se NPs synthesized using the leaf extract of *Urtica dioica* were well distributed and had a distinct spherical shape, with an average size of 162–85 nm, as represented with the SEM assay^36^^,24^. displayed that the Se NP synthesized using leaves extract of *Withania somnifera* were with greater numbers of the agglomerated spherical particles within the diameter range of 45–90 nm, as shown by the SEM images.

#### Transmission electron microscopy analysis

**Fig. 7:**
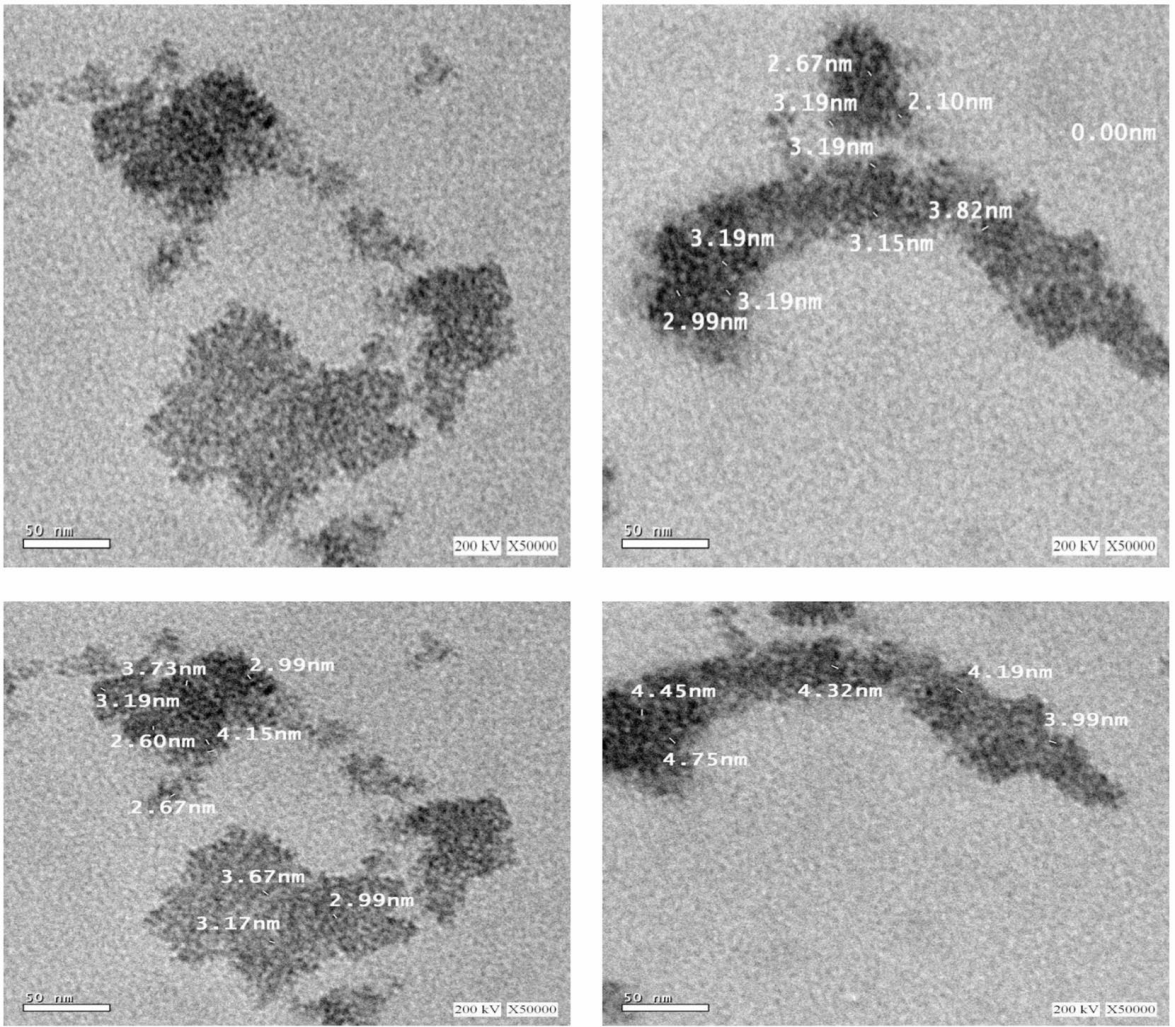
TEM images of Se NPs, ranging size of 2.60 nm to 4.75 nm.

The morphology and size of the synthesized selenium nanoparticles (Se NPs) were characterized using Transmission Electron Microscopy (TEM). As shown in Fig. 7, the Se NPs exhibit a quasi-spherical shape. TEM measurements indicate that particle size ranged from 2.60 to 4.75 nm, placing them within typical nanometer range of quantum dots. Their ultra-small size and associated quantum properties are expected to enhance their biological activity, particularly their antimicrobial potential. These findings are compared with previous study on green synthesis of selenium nanoparticles using *Diospyros montana* bark extract, which yielded particles ranging in size from 20 to 220 nm^45^, In contrast, our study demonstrates that green synthesis using *Olea europaea* extract produces significantly smaller nanoparticles (~ 4 nm), suggesting that *Olea europaea* leaf extract may be a more efficient reducing and stabilizing agent for nanoselenium synthesis.

Similar study by Hashem and Salem^36^ showed the development of polydispersed Se NPs *Urtica dioica* leaf extract with sizes ranging from 21.7 to 83.6 nm revealed by TEM investigation. Moreover, TEM analysis revealed the formation of spherical particles with average sizes ranging from 10.47 nm to 28.5 nm for Se NPs synthesized using *Moringa oleifera* leaf extract^46^. TEM image of the fabricated Se NPs prepared using *Aloe vera* leaf extract showed that the particles were monodispersed and spherical with mean particle size of 50 nm in diameter^47^.

### The antimicrobial activity of the biosynthesized Se NPs against different pathogenic strains

There are many metals and metal oxides nanoparticles as Se NPs have been documented as they have potential activity against many microbial agents. Many of the pathogenic organisms have developed antibiotic resistance due to the constant consumption of a broad range of antibiotics. So they have obtained resistance against most of the antimicrobial drugs^15^.

The biosynthesized Se NPs were applied in concentrations of 150, 75, and 50 µg/mL against the microbial strains, *S. aureus*,* E. coli*,* K. pneumoniae*,* C. freundii*, and *C. albicans.* The results in Table 1 and Supplementary Fig. 1 demonstrate the remarkable antimicrobial activity of Se NPs against all tested microbes at the used concentrations. Amikacin 30 µg was used as a positive control. The antimicrobial activity of Se NPs is directly proportional to the concentration, with the inhibitory activity of Se NPs gradually increasing as the Se NPs concentration increased. On the other hand, the *Olea europaea* leaf extract does not show any antimicrobial activity. Se NPs are mostly effective against *C. albicans* at 150 and 75 µg/mL, showing inhibition zone diameters of 35.98 ± 0.65 and 30.51 ± 0.84 mm, respectively. *C. freundii* and *S. aureus* record inhibition zone diameters of 33.65 ± 0.73 and 30.23 ± 0.12 mm, respectively, at 150 µg/mL. At 150 µg/mL *E. coli* and *K. pneumonia* are the least affected by Se NPs treatment, representing an inhibition zone diameters of 25.16 ± 0.36 and 27.33 ± 0.54 mm.

Compared the positive control Amikacin 30 µg, its antimicrobial efficiency is generally lower than that of Se NPs against the tested microbial strains. Amikacin 30 µg presents the highest antimicrobial activity against *C. freundii* and *E.coli*, recording inhibition zone diameters of 22 ± 0.10 and 20.32 ± 0.45 mm, respectively. On the other hand, it shows moderate antimicrobial effect against *S. aureus*, *K. pneumonia*, and *C. albicans*, with the inhibition zone diameters of 19.5 ± 0.37, 18.16 ± 0.12, and 18.53 ± 0.72 mm, respectively.

The MIC and MBC of Se NPs were measured at values ranging from 3.125 to 100 µL as presented in Table 1. The results indicate that the MIC of Se NPs toward *S. aureus* is 25 µg/mL and MBC is 50 µL, while the MICs assessment of Se NPs toward *E. coli* and *K. pneumonia* are 50 µg/mL and MBCs are 100 µg/mL. The MICs assessment of Se NPs toward *C. freundii* and *C. albicans* are 12.5, 6.25 µg/mL. MBC of Se NPs against *C. freundii* is 25 µg/mL, and MFC of Se NPs against *C. albicans*is 12.5 µg/mL. Se NPs have two unique behaviors: being bacteriostatic at fewer concentrations and bactericidal at higher levels. Similarly^48^, reported that the minimum inhibitory concentration (MIC) of *Aspergillus quadrilineatus* and *Aspergillus ochraceus*-synthesized Se NPs against *C. albicans*, the most susceptible fungal pathogen to all Se NPs, was 62.5 µg/mL. Also, twenty pathogenic methicillin-resistant *Staphylococcus aureus* (MRSA) strains were shown to be sensitive to phyto-synthesized Se NPs’ antibacterial effects and demonstrating low MIC ranging 50 to 800 µg/mL^49^.

**Table 1:** The antimicrobial activity of the biosynthesized Se NPs represented as inhibition zone diameter (mm).

The pathogenic strains	Se NPs conc. (µg/mL)			Amikacin 30 µg	MIC (µg/mL)	MBC/MFC (µg/mL)
	150	75	50			
*S. aureus*	30.23 ± 0.12	28.0 ± 0.21	25.78 ± 0.34	19.5 ± 0.37	25	50
*E. coli*	25.16 ± 0.36	23.15 ± 0.64	6.0 ± 0.0	20.32 ± 0.45	50	100
*K. pneumoniae*	27.33 ± 0.54	24.32 ± 0.54	18.14 ± 0.36	18.16 ± 0.12	50	100
*C. freundii*	33.65 ± 0.73	29.45 ± 0.71	25.0 ± 0.64	22 ± 0.10	12.5	25
*C. albicans*	35.98 ± 0.65	30.51 ± 0.84	26.71 ± 0.13	18.53 ± 0.72	6.25	12.5

Nanoparticles may adhere to bacterial membranes due to their high surface area and distinct physicochemical properties^50^. The Se NPs can be used alone or combined with other medical drugs to act against several of fungal and bacterial species, despite of the method of synthesis^14^. As described by (Blinov et al., 2025), the antimicrobial activity of Se NPs has many mechanisms of action as degradation of proteins, cell wall, and membrane damage. The bonding of selenium ions with -SH, -NH, or -COOH functional groups of microbial proteins causes them to lose their tertiary and quaternary structure and activities. Furthermore, the antimicrobial activity of Se NPs may result from the inhibition of ion and nutrient transport across cell walls, thereby disrupting vital processes within the microbial cell. Se NPs causes a photocatalytic effect against bacteria, and an increase in the production of reactive oxygen species (ROS) leading to endogenous ATP depletion (Fahmy et al., 2025). Se NPs may inhibit the action of the dehydrogenase enzyme, in addition to destroying the integrity of the cell membrane, and finally inhibit the ability of bacteria to form bacterial films^51^. Alternatively, nanoparticles may interact with bacterial membranes through hydrophobic and electrostatic interactions, potentially entering cells via pinocytosis. Smaller nanoparticles can enhance membrane permeability, leading to intracellular leakage and, subsequently, apoptosis^12^.

Se NPs’ antibacterial effect against microbes is determined by their concentration, treatment period, and microbiological species. The observation that sensitivity increases at high concentrations of selenium nanoparticles can be attributed to the presence of large numbers of selenium ions, which are directly related to enhancing the level of microbial cell stress, leading to their death^52^. NPs’ antimicrobial action is related to cell wall structure, as Gram-positive strains comprise of thick layers of peptidoglycan, whereas Gram-negative strains are composed of thin layers. This difference influences the diffusion of NPs within cells^53^. The thick peptidoglycan layer prevents and delays the diffusion of Se NPs, so Gram-positive have less antibacterial than Gram-negative bacteria^54^. This causes a significant accumulation of Se NPs on Gram-positive bacteria cell surface, facilitating cell death. Se NPs’ efficacy against various strains of *Candida* is attributed to their ability to disrupt the sterol composition in the *Candida* cell wall by delaying the ergosterol production pathway^55^.

### The antibiofilm activity of Se NPs against pathogenic strains

**Fig. 8:**
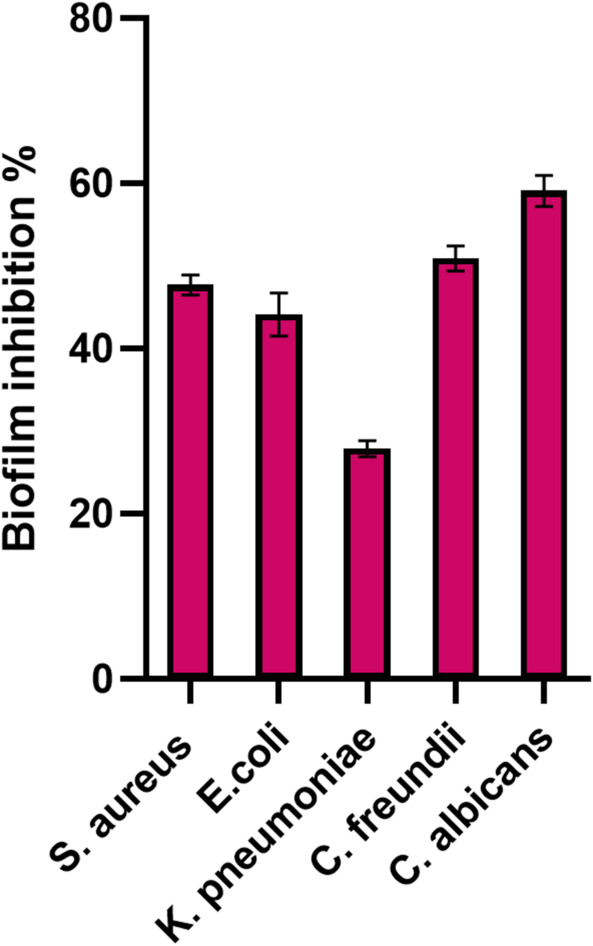
The antibiofilm activity of the biosynthesized Se NPs against the tested microbial strains.

Using the crystal violet tube assay, the antibiofilm activity of Se NPs was assessed at λ = 570 nm against *S. aureus*,* E. coli*,* K. pneumoniae*,* C. freundii*, and *C. albicans*. The antibiofilm efficiency of Se NPs is shown in Fig. 8.

At 75 µg/mL concentration, Se NPs exhibit a superior percentage of biofilm inhibition against all the tested microbes. The greatest biofilm inhibition parentage is registered for *C. albicans* (59.04 ± 1.8700%), followed by *C. freundii*,* S. aureus*, and *E. coli* with biofilm inhibition percentage of 50.90 ± 1.56, 47.66 ± 1.23, and 44.07 ± 2.65% respectively. On the other hand, *K. pneumoniae* exhibits the lowest biofilm inhibition percentage of 27.82 ± 0.98%.

The extracellular polymeric substances (EPS) of biofilms can be composed of various polysaccharides, proteins, lipids, nucleic acids, and lipopolysaccharides which contribute to bacterial antibiotic resistance. The viscosity of the EPS matrix, cell density, liquid flow, and pore properties of water spaces influence how nanoparticles are transported into biofilms. Additionally, EPS is crucial for maintaining the structure of the biofilm^56^.

There are three mechanisms through which nanoparticles (NPs) interact with biofilms: they reach the biofilm surface at the biofilm-fluid interface, attach to the outer layer of the biofilm, and facilitate easier penetration into the deeper structures of the biofilm. Se NPs disrupt or damage this layer, rendering biofilms more susceptible to penetration (Afrasiabi and Partoazar, 2024). The hydrophobicity of cell surfaces (CSH) influences the adhesion process of bacteria to solid surfaces by enhancing attachment strength and decreasing repulsive forces between the surfaces and bacteria during the adhesion phase. Nanoparticles (NPS) diminish the attachment of biofilms, thereby reducing their formation^57^. Additionally, the physicochemical properties of nanoparticles play a crucial role, as factors like surface charge, hydrophobicity, and a high surface-to-volume ratio facilitate diffusion and penetration into biofilms^58^. By influencing bacterial cell-cell communication or inhibiting the quorum sensing (QS) signaling system, NPs work against the microbial cell’s QS. This prevents the formation of various signaling molecules and the synthesis of molecule-receptor complexes^59^.

## Conclusion

Se NPs were synthesized using the aqueous leaf extract of *Olea europaea* in a green approach. Their characterization was verified using a UV-Visible spectrophotometer, FTIR, SEM, EDX, and TEM. Se NPs were evenly distributed, quasi-spherical, and size from 2.60 to 4.75 nm. The biosynthesized Se NPs were tested at concentrations of 150, 75, and 50 µg/mL against the microbial strains, *S. aureus*,* E. coli*,* K. pneumoniae*,* C. freundii*, and *C. albicans*. Se NPs have mostly inhibited the growth of the tested strains rather than the other concentrations. At 150 µg/mL, Se NPs have mostly inhibited the growth of *C. albicans*, displaying an inhibition zone diameter of 35.98 ± 0.65 mm, followed by *C. freundii* and *S. aureus* that registered inhibition zone diameters of 33.65 ± 0.73 and 30.23 ± 0.12 mm, respectively. However, *E. coli* and *K. pneumonia* were less inhibited displaying inhibition zone of 25.16 ± 0.36 and 27.33 ± 0.54 mm. Se NPs possessed excellent antibiofilm activities with biofilm inhibition percentage for *C. albicans* (59.04 ± 1.87%) while the lowest biofilm inhibition percentage of 27.82 ± 0.98% was reported for *K. pneumoniae*. These results suggested that the green-synthesized Se NPs may be utilized as antibacterial and antibiofilm agents against various pathogenic bacterial strains. Limiting points that need further study are the increasing number of tested pathogenic fungi and the thermodynamic stability of Se NPs. Also, more investigations such as cytotoxicity and antioxidant are needed.

## Supplementary Information

Below is the link to the electronic supplementary material.

Supplementary Material 1

## Data Availability

The data and materials supporting the study’s conclusions are accessible from the corresponding author upon request.

## References

[1] Wani, M. Y., Wani, I. A. &amp; Rai, A. *Nanotechnology Based Strategies for Combating Antimicrobial Resistance* (Springer, 2024).

[2] Munita, J. M. &amp; Arias, C. A. Mechanisms of antibiotic resistance. In *Virulence Mechanisms of Bacterial Pathogens* 481–511 (2016).

[3] Ntallis, C., Martin, N. I., Edwards, A. M. & Weingarth, M. Bacterial cell envelope-targeting antibiotics. *Nat. Rev. Microbiol.* **2025**, 1–14 (2025). 10.1038/s41579-025-01247-x 41083887

[4] Heesterbeek, D. et al. Complement-dependent outer membrane perturbation sensitizes Gram-negative bacteria to Gram-positive specific antibiotics. *Sci. Rep.* **9**, 3074 (2019). 30816122 10.1038/s41598-019-38577-9 PMC6395757

[5] Turner, J. et al. The chemical relationship among beta-lactam antibiotics and potential impacts on reactivity and decomposition. *Front. Microbiol.* **13**, 807955 (2022). 35401470 10.3389/fmicb.2022.807955 PMC8988990

[6] Owen, R. et al. Olives and olive oil in cancer prevention. *Eur. J. Cancer Prev.* **13**, 319–326 (2004). 15554560 10.1097/01.cej.0000130221.19480.7e

[7] Koca, U., Süntar, I., Akkol, E. K., Yılmazer, D. & Alper, M. Wound repair potential of *Olea europaea* L. leaf extracts revealed by in vivo experimental models and comparative evaluation of the extracts’ antioxidant activity. *J. Med. Food*. **14**, 140–146 (2011). 21128831 10.1089/jmf.2010.0039

[8] Hussein, E. M. A. & Fathy, R. M. Optical, structural, and antifungal properties of nanosilver borate bioactive glasssynthesized using gamma rays on the survival of Candida albicans and Candida tropicalis. *J. Biotechnol.* **410**, 33–44 (2026). 41276133 10.1016/j.jbiotec.2025.11.013

[9] Khalil, K. A., Fouad, H., Elsarnagawy, T. & Almajhdi, F. N. Preparation and characterization of electrospun PLGA/silver composite nanofibers for biomedical applications. *Int. J. Electrochem. Sci.* **8**, 3483–3493 (2013).

[10] Truong, L. B., Medina-Cruz, D., Mostafavi, E. & Rabiee, N. Selenium nanomaterials to combat antimicrobial resistance. *Molecules* **26**, 3611 (2021). 34204666 10.3390/molecules26123611 PMC8231168

[11] Rao, H. et al. Approaches for mitigating microbial biofilm-related drug resistance: a focus on micro-and nanotechnologies. *Molecules* **26**, 1870 (2021). 33810292 10.3390/molecules26071870 PMC8036581

[12] Patil, B. N. & Taranath, T. C. *Limonia acidissima* L. leaf mediated synthesis of silver and zinc oxide nanoparticles and their antibacterial activities. *Microb. Pathogen*. **115**, 227–232 (2018). 29248515 10.1016/j.micpath.2017.12.035

[13] Sokary, R., Raslan, H. A. & Fathy, R. M. Green synthesis of CdS/flaxseed mucilage nanocomposite films using gamma irradiation for packaging applications. *Radiochim. Acta* **112**, 427–444 (2024).

[14] Oliveira, T. P. S., Lima, A. K. O. & Muehlmann, L. A. An updated review of the antimicrobial potential of selenium nanoparticles and selenium-related toxicological issues. *Future Pharmacol.* **5**, 3 (2025).

[15] Bisht, N., Phalswal, P. & Khanna, P. K. Selenium nanoparticles: A review on synthesis and biomedical applications.Mater. *Adv* **3** (3), 1415–1431 (2022).

[16] Ferro, C., Florindo, H. F. & Santos, H. A. Selenium nanoparticles for biomedical applications: from development and characterization to therapeutics. *Adv. Healthc. Mater.* **10** (16), 2100598 (2021). 10.1002/adhm.202100598 34121366

[17] Fathy, R. M., Salem, M. S. E. & Mahfouz, A. Y. Biogenic synthesis of silver nanoparticles using Gliocladium deliquescens and their application as household sponge disinfectant. *Biol. Trace Elem. Res.* **196** (2), 662–678 (2020). 31808109 10.1007/s12011-019-01958-2

[18] El-Batal, A. et al. Impact of silver and selenium nanoparticles synthesized by gamma irradiation and their physiological response on early blight disease of potato. *J. Chem. Pharm. Res.* **8** (4), 934–951 (2016).

[19] Sagheer, R., Nasibullah, M. &amp; Iqbal, N. Recent trends in antimicrobial drug resistance and implications for the needs of microbial toxicology research. In *Antimicrobial Resistance in Agriculture and its Consequences* 131–156 (CRC, 2024). .

[20] Sans-Serramitjana, E. et al. Antimicrobial activity of selenium nanoparticles (Se NPs) against potentially pathogenic oral microorganisms: a scoping review. *Pharmaceutics* **15**, 2253 (2023). 37765222 10.3390/pharmaceutics15092253 PMC10537110

[21] Ndwandwe, B. K., Malinga, S. P., Kayitesi, E. & Dlamini, B. C. Advances in green synthesis of selenium nanoparticles and their application in food packaging. *Int. J. Food Sci. Technol.* **56**, 2640–2650 (2021).

[22] Afonso, I. S. et al. Green synthesis of nanoparticles from olive oil waste for environmental and health applications: Areview. *J. Environ. Chem. Eng.* **12** (5), 114022 (2024).

[23] Sarkar, R. D., Lahkar, P. & Kalita, M. C. Glycosmis pentaphylla (Retz.) DC leaf extract mediated synthesis of selenium nanoparticle and investigation of its antibacterial activity against urinary tract pathogens. *Bioresour. Technol. Rep.* **17**, 100894 (2022).

[24] Alagesan, V. & Venugopal, S. Green synthesis of selenium nanoparticle using leaves extract of Withania somniferaand its biological applications and photocatalytic activities. *Bionanoscience* **9** (1), 105–116 (2019).

[25] Abd-El-Aziz, A. S. et al. Production and characterization of melanin pigment from black fungus Curvularia soli AS21ON076460 assisted gamma rays for promising medical uses. *Microb. Cell. Fact.* **23** (1), 68 (2024). 38408972 10.1186/s12934-024-02335-y PMC10895916

[26] Kodous, A. S., Fathy, R. M., Mazhar, A., Shafaa, M. W. & Shalaby, M. Green synthesis of β-carotene-loaded liposome as antibacterial, antibiofilm and anti-inflammatory modulator: targeting NO/iNOS/NF-κB signaling pathway in MCF-7 cancer cell line. *Process. Biochem.* **153**, 135–153 (2025).

[27] El-Nemr, K. F. et al. Polyvinyl alcohol/gelatin irradiated blends fi lled by lignin as green filler for antimicrobial packaging materials. *Int. J. Environ. Anal. Chem.* **100** (14), 1578–1602 (2020).

[28] Štefanková, D., Skrbek, K., Pižl, M. & Bartůněk, V. Nano and mesosized selenium and its synthesis using the ascorbic acid route. *J. Non-Cryst Solids*. **616**, 122462 (2023).

[29] Siddiqui, S. A. et al. Effect of selenium nanoparticles on germination of *Hordéum Vulgáre* barley seeds. *Coatings* **11**, 862 (2021).

[30] Hassanien, R., Abed-Elmageed, A. A. & Husein, D. Z. Eco‐friendly approach to synthesize selenium nanoparticles: photocatalytic degradation of sunset yellow azo dye and anticancer activity. *ChemistrySelect* **4**, 9018–9026 (2019).

[31] Mali, S. C., Dhaka, A., Githala, C. K. & Trivedi, R. Green synthesis of copper nanoparticles using *Celastrus paniculatus* Willd.leaf extract and their photocatalytic and antifungal properties. *Biotechnol. Rep.* **27**, e00518 (2020). 10.1016/j.btre.2020.e00518 PMC7475076 32923378

[32] Ying, S. et al. Green synthesis of nanoparticles: current developments and limitations. *Environ. Technol. Innov.* **26**, 102336 (2022).

[33] Shahbaz, M. et al. Antifungal activity of green synthesized selenium nanoparticles and their effect on physiological, biochemical, and antioxidant defense system of mango under mango malformation disease. *PLoS One*. **18**, e0274679 (2023). 36749754 10.1371/journal.pone.0274679 PMC9904489

[34] Fouda, A. et al. Antimicrobial, antiviral, and in-vitro cytotoxicity and mosquitocidal activities of Portulaca oleracea-based green synthesis of selenium nanoparticles. *J. Funct. Biomater.* **13** (3), 157 (2022). 36135592 10.3390/jfb13030157 PMC9504135

[35] Ullah, A. et al. Biosynthesis of selenium nanoparticles (via *Bacillus subtilis* BSN313), and their isolation, characterization, and bioactivities. *Molecules* **26**, 5559 (2021). 34577029 10.3390/molecules26185559 PMC8468162

[36] Hashem, A. H., Salem, S. & S. Green and ecofriendly biosynthesis of selenium nanoparticles using *Urtica dioica* (stinging nettle) leaf extract: antimicrobial and anticancer activity. *Biotechnol. J.* **17**, 2100432 (2022). 10.1002/biot.202100432 34747563

[37] Sheikhalipour, M. et al. Chitosan–selenium nanoparticle (Cs–Se NP) foliar spray alleviates salt stress in bitter melon. *Nanomaterials* **11**, 684 (2021). 33803416 10.3390/nano11030684 PMC7999252

[38] Garza-García, J. J. et al. Selenium nanoparticles based on Amphipterygium glaucum extract with antibacterial, antioxidant, and plant biostimulant properties. *J. Nanobiotechnol.* **21** (1), 252 (2023). 10.1186/s12951-023-02027-6 PMC10399041 37537575

[39] Shanlee, S. S. R., Ragumoorthy, C., Chen, S. M. & Stella, P. C. R. Electrochemical profiling of bendiocarb residues in food matrices using an interfacially engineered halloysite-ternary hydroxide hybrid. *Process. Saf. Environ. Prot.* **2025**, 107254 (2025).

[40] Baran, A. Environmentally friendly rapid green synthesis of SeNPs using grapefruit (Citrus paradisi) leaves extract, and their antimicrobial potential. *Int. J. Agric. Environ. Food Sci.* **8** (2), 315–326 (2024).

[41] Iqbal, M. F. et al. Comparative study of the ability of green synthesized Se-NPs and CTS-NPs to overcome drought stress in *Oryza sativa* L. for regenerative nanoengineering in agriculture. *New. J. Chem.* **49**, 7358–7375 (2025).

[42] Tamboli, A. M. M. & Tade, J. M. Zeta potential: a comprehensive review. *Int. Res. J. Pharm. Med. Sci.* **8**, 115–124 (2025).

[43] Verma, P. & Maheshwari, S. K. Preparation of sliver and selenium nanoparticles and its characterization by dynamic light scattering and scanning electron microscopy. *J. Microsc. Ultrastruct.* **6**, 182–187 (2018). 30464890 10.4103/JMAU.JMAU_3_18 PMC6206752

[44] Mishra, M., Shukla, N., Fatima, M. & Singh, N. K. Biogenic selenium nanoparticles as nanopriming agents: promoting germination and strengthening antioxidant defense in rice (*Oryza sativa* L). *Biocatal. Agric. Biotechnol.* **65**, 103568 (2025).

[45] Puri, A. et al. Plant-derived selenium nanoparticles: investigating unique morphologies, enhancing therapeutic uses, and leading the way in tailored medical treatments. *Mater. Adv.* **5**, 3602–3628 (2024).

[46] Olaoye, A. B., Owoeye, S. S. & Nwobegu, J. S. Facile green synthesis of plant-mediated selenium nanoparticles (SeNPs) using *Moringa oleifera* leaf and bark extract for targeting α-amylase and α-glucosidase enzymes in diabetes management. *Hybrid. Adv.* **7**, 100281 (2024).

[47] Fardsadegh, B. & Jafarizadeh-Malmiri, H. Aloe vera leaf extract mediated green synthesis of selenium nanoparticlesand assessment of their in vitro antimicrobial activity against spoilage fungi and pathogenic bacteria strains. *GreenProcess Synth.* **8** (1), 399–407 (2019).

[48] Hussein, H. G., El-Sayed, E. S. R., Younis, N. A., Hamdy, A. E. H. A. & Easa, S. M. Harnessing endophytic fungi for biosynthesis of selenium nanoparticles and exploring their bioactivities. *AMB Express*. **12**, 68 (2022). 35674975 10.1186/s13568-022-01408-8 PMC9177918

[49] Ibrahim, Z. K. & Al-Haideri, H. H. Phyto-synthesize of selenium nanoparticles via *Psidium guajava* leaves and study the efficacy versus Methicillin-Resistant Staphylococcus aureus (MRSA) and their biofilm. *Microb. Biosyst J.* **10**, 221–231 (2025).

[50] Patil, B. N., Taranath, T. C. & Konkal, P. Antimycobacterial and antibacterial activity of green-synthesized silver and zinc oxide nanoparticles using *Diospyros montana* L. leaf extract. *Next Nanotechnol*. **8**, 100248 (2025).

[51] Kumar, N., Thorat, S. T., Patole, P. B., Gite, A. & Reddy, K. S. Protective role of selenium and selenium-nanoparticles against multiple stresses in *Pangasianodon hypophthalmus*. *Fish. Physiol. Biochem.* **50**, 239–258 (2024). 37656302 10.1007/s10695-023-01231-3

[52] Rani, B., Shanmugam, R. & Govindharaj, S. Green synthesis and biomedical applications of selenium nanoparticles and its based nanocomposite-a review. *Test. Psychom Methodol. Appl. Psychol.* **32**, 1146–1152 (2025).

[53] Mahfouz, A. Y., Abed, N. N., Abd-EL-Aziz, A. S. & Fathy, R. M. Green synthesis of gamma rays-induced melanin-based bismuth oxide nanoparticles for evaluation of the antibacterial and anti-virulence activities against extra-intestinal pathogenic bacteria. *World J. Microb. Biotechnol.* **41**, 1–18 (2025). 10.1007/s11274-025-04533-1 PMC12380928 40856931

[54] Yasir, R. M. & Zaki, N. H. Antioxidant and anticancer activities of bio-synthesized selenium nanoparticles by *Escherichia coli*. *Asian Pac. J. Cancer Prev.* **26**, 1303 (2025). 40302083 10.31557/APJCP.2025.26.4.1303 PMC12227980

[55] Mohamed, E. A. & El–Zahed, M. M. Anticandidal applications of selenium nanoparticles biosynthesized with *Limosilactobacillus fermentum* (OR553490). *Discov. Nano*. **19**, 115 (2024). 38980559 10.1186/s11671-024-04055-z PMC11233486

[56] Saharan, B. S., Beniwal, N. & Duhan, J. S. From formulation to function: a detailed review of microbial biofilms and their polymer-based extracellular substances. *Microbe* **5**, 100194 (2024).

[57] Zhu, S., Li, M., Qian, T., Chen, J. & Pan, T. Influence of surfactants on interfacial microbial degradation of hydrophobic organic compounds. *Catalysts* **15**, 187 (2025).

[58] Wang, C., Shahriar, S. S., Su, Y. & Xie, J. Versatile nanomaterials used in combatting biofilm infections. *Nanomedicine* **20**, 501–518 (2025). 39887017 10.1080/17435889.2025.2459049 PMC11875486

[59] Juszczuk-Kubiak, E. Molecular aspects of the functioning of pathogenic bacteria biofilm based on quorum sensing (QS) signal-response system and innovative non-antibiotic strategies for their elimination. *Int. Int. J. Mol. Sci.* **25**, 2655 (2024). 38473900 10.3390/ijms25052655 PMC10931677

