# Supplementary material for: Facile green synthesis of selenium nanoparticles using olive (Olea europaea) leaf extract and their antimicrobial and antibiofilm properties
Source: Sci Rep. 2026 May 16;16:15224. doi: 10.1038/s41598-026-47329-5 (PMC13179952; doi:10.1038/s41598-026-47329-5)
Supplement: Supplementary file 1 — Supplementary Material 1 [file 41598_2026_47329_MOESM1_ESM.docx]

| **A**  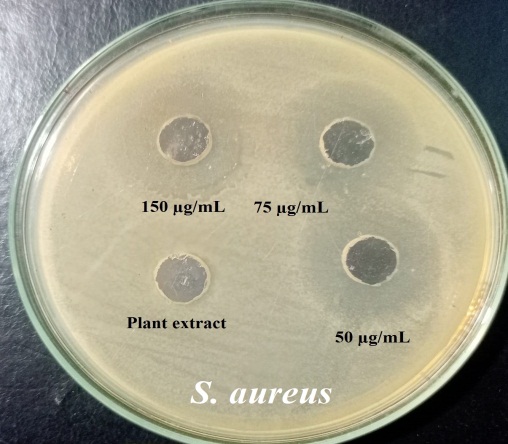 | **A'**  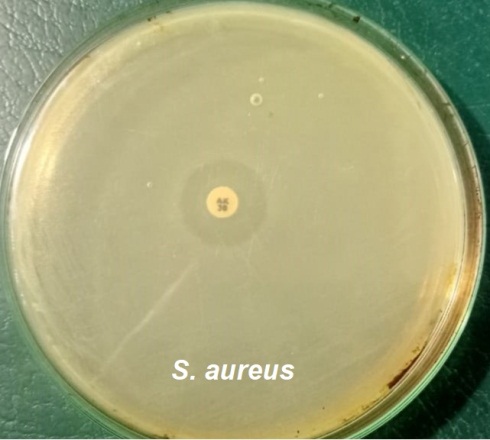 |
| --- | --- |
| **B**  **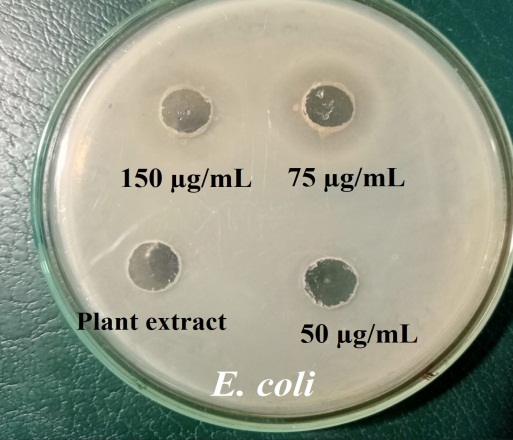** | **Bˋ**  **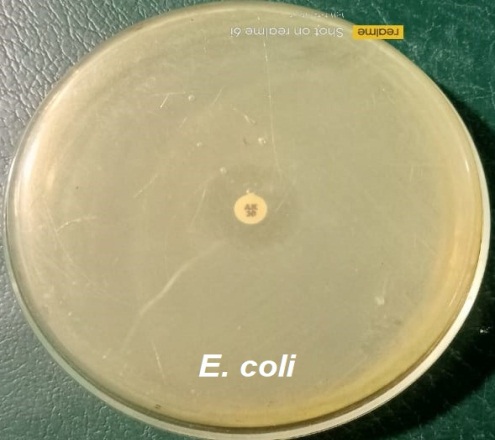** |
| **C**  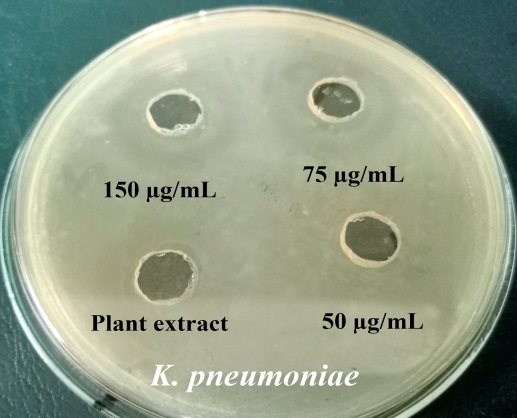 | **Cˋ**  **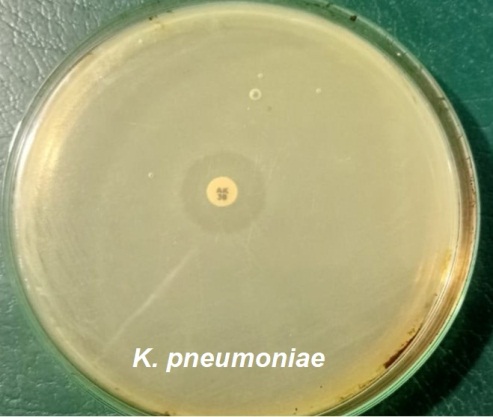** |
| **D**  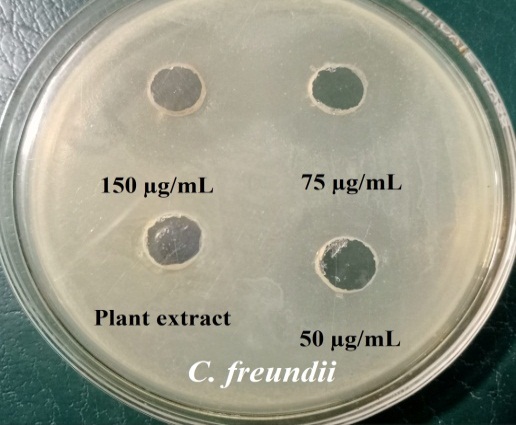 | **Dˋ**  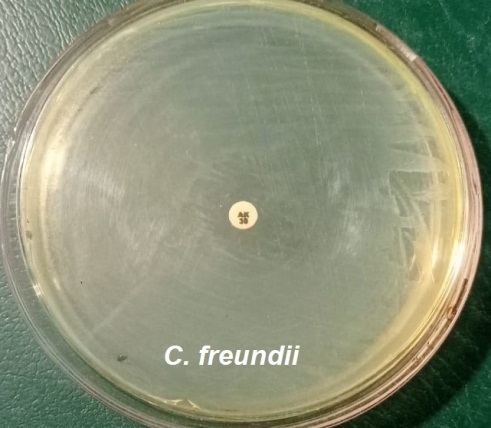 |
| **E**  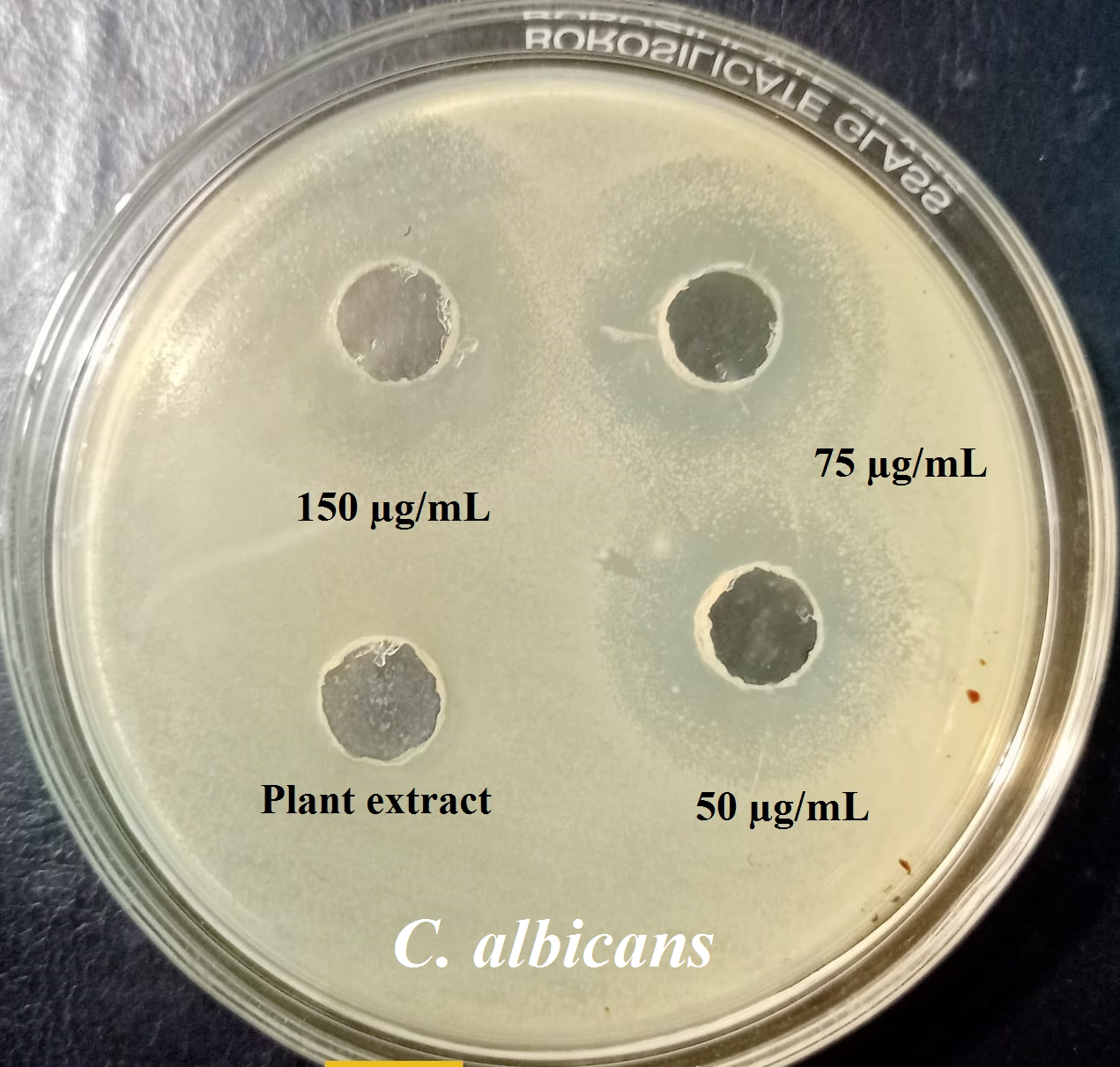 | **Eˋ**  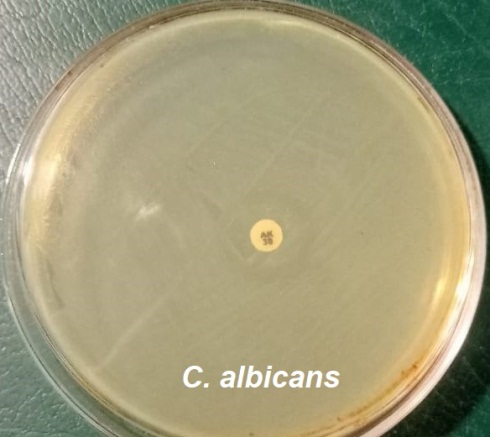 |

**Fig 1. Antimicrobial activity of the biosynthesized Se NPs at concentrations of 50, 75, and 150 µg/mL compared to the negative control plant extract and the positive control Amikacin 30 µg against strains A, Aˋ) *S. aureus,* B, B)** ***E. coli,* C, Cˋ) *K. pneumoniae,* D, Dˋ) *C. freundii,* and E, Eˋ) *C. albicans***
